# Safety and Efficacy of Low Molecular Weight Heparin for Thromboprophylaxis in the Elderly: A Network Meta-Analysis of Randomized Clinical Trials

**DOI:** 10.3389/fphar.2021.783104

**Published:** 2021-12-10

**Authors:** Hui-qin Yang, Man-cang Liu, Wen-jun Yin, Ling-yun Zhou, Xiao-cong Zuo

**Affiliations:** ^1^ Department of Pharmacy, The Third Xiangya Hospital, Central South University, Changsha, China; ^2^ Center of Clinical Pharmacology, The Third Xiangya Hospital, Central South University, Changsha, China

**Keywords:** the elderly, low molecular weight heparins, venous thrombus embolism, network meta-analysis, bleeding

## Abstract

**Background:** Given their changing pathophysiology, elderly patients carry a high risk of embolism and bleeding events; hence, use of appropriate anticoagulants is very important. Low molecular weight heparin (LMWH) is one of the most widely used anticoagulants although LMWHs differ in their anti-Xa, antithrombin, and anticoagulant activities. To date, no study has directly compared the safety and efficacy of different LMWHs in the elderly. We aimed to compare such differences by conducting a network meta-analysis.

**Methods:** We searched the Pubmed, Embase, and Cochrane databases for randomized controlled trials (RCTs) of LMWHs that included patients ≥60 years old up to July 22, 2020. Safety outcomes included venous thromboembolism (VTE) or VTE-related death, deep thrombus embolism, and pulmonary embolism. Safety outcomes were clinically relevant bleeding, major bleeding, minor bleeding, and all-cause death. We calculated relative ratios (RR) and 95% confidence intervals (CI) for all outcomes. The cumulative ranking probabilities (SUCRA) were conducted to rank the comparative effects and safety of all LMWHs.

**Results:** We included 27 RCTs (30,441 elderly), comprising five LMWHs. LMWH was more effective than placebo in preventing VTE or VTE-related death (RR 0.36, 95% CI 0.25–0.53) but less effective than a novel oral anticoagulant (RR 1.59, 95% CI 1.33–1.91) and safer than acenocoumarol regarding risk of clinically relevant bleeding (RR 0.67, 95% CI 0.49–0.90). However, indirect comparison of efficacy and safety of the five LMWHs showed no significant difference in our network analysis, and the subgroup analyses (such as in patients with deep venous thrombosis, cardiac disease, or age >65 years old) supported the results. The SUCRA showed that tinzaparin performed best in preventing VTE or VTE-related death (SUCRA 68.8%, cumulative probability 42.3%) and all-cause death (SUCRA 84.2%, cumulative probability 40.7%), whereas nadroparin was predominant in decreasing the risk of clinically relevant bleeding (SUCRA 84.8%, cumulative probability 77.0%).

**Conclusions:** On present evidence, there are no significant differences in the efficacy and safety of different LMWHs for the elderly. According to the rank probability analysis, nadroparin seems to be safer for the elderly with a high risk of bleeding, whereas tinzaparin is more effective for those with low bleeding risk.

## Introduction

The number of elderly individuals continues to grow rapidly worldwide. In China, for instance, the proportion of older adults (≥65 years) is expected to rise from 12.6% of the total population (more than 176 million) in 2019 to 26.9% in 2050 (Yang et al., 2020). Venous thromboembolism (VTE) is the leading preventable cause of death in hospitalized patients (Macdougall and Spyropoulos, 2021) and the second leading cause of malignancy-related mortality (Xiong, 2021). The incidence of VTE increases exponentially with age, rising from 1/10,000 in people aged 25–30 years to 8/1000 in those ≥85 years (Palareti and Poli, 2018). This increase can be explained by pathophysiological changes in the elderly, often associated with diverse diseases, multiple medications, and other risk factors for VTE as well as possible age-related liver and kidney dysfunction, endothelial dysfunction, inflammation, frailty, and immobility (Zhang et al., 2021); (Kozek-Langenecker et al., 2018). In addition to the higher risk of VTE, these changes also increase the risk of bleeding in older persons, and the use of anticoagulants greatly increases the risk (Montalto et al., 2020). Hence, an appropriate anticoagulant treatment is particularly important for elderly patients.

Low molecular weight heparin (LMWH) is widely used in the clinic and even recommended by a European guideline for elderly patients with renal failure or during the perioperative period of knee or hip replacement with no restrictions on specific types (Kozek-Langenecker et al., 2018). LMWH is a general term for a class of LMWH prepared by depolymerization of unfractionated heparin (UFH) with a mean molecular weight of 5000 Da (Sharif-Askari et al., 2014). With an enhanced anti-Xa: IIa ratio, LMWH may provide more therapeutic benefit than UFH (Antman and Handin, 1998) and a predictable anticoagulant effect for almost all populations (Samama, 2011). In fact, LMWHs are different in their biodynamic patterns, efficacy, and safety because of their different manufacturing processes, molecular weights, anti-Xa, antithrombin, and anticoagulant activities (Frydman, 1996).

Although several studies explore the pharmacokinetics of LMWHs in the elderly, the efficacy and safety of different LMWHs in this population remain unclear yet. Enoxaparin and nadroparin are reported to accumulated significantly in the elderly (Mismetti et al., 1998; Mahe et al., 2007), and enoxaparin dose adjustment based on renal function can decrease the risk of bleeding (Pellizzari et al., 2018), whereas the monitoring of anti-Xa activity is not necessary (Berges et al., 2007). The pharmacokinetics of tinzaparin and bemiparin show no significant difference between elderly and young healthy volunteers, and dose adjustment was not required in the elderly (Mahe et al., 2007). In fact, an earlier study indicates that tinzaparin did not require dose adjustment even in elderly individuals with renal insufficiency (Siguret et al., 2000). Thus, LMWHs appear to differ in their efficacy/safety ratio because of these inherent differences, whereas no clinical study has directly compared their efficacy and safety in the elderly.

We aimed to compare the efficacy and safety of different LMWHs in the elderly by conducting a network meta-analysis to provide better anticoagulation options for older adults as it is unclear whether there are differences in the efficacy and safety of different LMWHs and the specific needs of the elderly regarding anticoagulants.

## Materials and Methods

### Search Strategy and Selection Criteria

Our research protocol was registered with the International Prospective Register of systematic reviews (PROSPERO, CRD42021241699). Two authors independently searched Pubmed, Embase, and Cochrane to identify studies that compared the efficacy or safety of LMWHs with other treatment in the elderly (from inception to July 22, 2020). The following search terms were included: “low-molecular-weight,” “LMWH,” “nadroparin,” “enoxaparin,” “dalteparin,” and many other generic and trade names of LMWHs; “elderly,” “aged,” “elder people,” “old people,” “the old,” “old man,” and “aging.” The search details are provided in Supplementary Tables S1–S3. We also evaluated the bibliographies of published studies. The cutoff age (60 years old) for the elderly is defined by the World Health Organization (World Health Organization, 1989; Zhang et al., 2020). Studies were considered eligible if they fulfilled the following criteria: 1) studies that included patients aged ≥60 years; 2) interventions that were specific kinds of LMWHs, and the control group were unrestricted; 3) randomized controlled trials (RCTs); and 4) studies published in English. Exclusion criteria were 1) studies that did not report the interest endpoints; 2) the full-text version could not be acquired online; and 3) studies that were not relevant.

### Outcome Measures

Efficacy endpoints included incidence of VTE or VTE-related death, deep venous thrombosis (DVT), and pulmonary embolism (PE). VTE or VTE-related death was defined as asymptomatic proximal DVT, symptomatic proximal DVT, symptomatic distal DVT, symptomatic nonfatal PE, or VTE-related death; for studies reporting only DVT or PE, the same data were also used for VTE or VTE-related death. DVT and PE were defined by the included studies (Fuentes et al., 2019). Safety endpoints included clinically relevant bleeding, major bleeding, minor bleeding, and all-cause death. Clinically relevant bleeding was defined as all bleeding events, and the data were used for clinically relevant bleeding when studies reported only major bleeding. Major and minor bleeding were defined according to the International Society on Thrombosis and Hemostasis criteria (Schulman and Kearon, 2005); All-cause death included VTE-related death and death for other reasons. The definition of outcomes in original RCTs is provided in Supplementary Table S5.

### Study Selection

Two researchers (Yang and Zhou) screened the studies independently. Studies were preliminarily screened according to the title and abstract and finally included or excluded according to the full text. Studies were included only if they met the inclusion criteria (data were also collected from some subgroups of RCTs, meta-analysis, and pooled analyses). If there was any disagreement, another author also independently evaluated the studies, after which the decisions were finalized through a group discussion.

### Data Extraction, Quality Assessment, and Bias Evaluation

A unified data extraction form was used to extract the relevant data from engaged RCTs, including 1) characteristics of the studies: title, first author, year of publication, country, number of centers, treatment, study object, sample size, and so on; 2) patient characteristics: age, gender, weight, and definition of the elderly; 3) interest endpoints: VTE or VTE-related death, DVT, PE, clinically relevant bleeding, major bleeding, minor bleeding, and all-cause death. If the related events were reported as a percentage, the figure was rounded.

The study quality of publications included in this analysis was evaluated by two researchers using the Cochrane Collaboration’s risk of bias tool, which evaluated seven possible biases: random sequence generation, allocation concealment, blinding of participants and personnel, blinding of outcome assessments, incomplete outcome data, selective outcome reporting, and other potential bias. The potential for publication bias was assessed by Begg funnel plots.

### Statistical Analysis

All of our network analysis was performed using the STATA statistical software (version 13.0). Node-splitting analysis was used to calculate the inconsistencies of our models. Pooled results of direct and indirect comparisons were reported as relative ratios (RR) and 95% confidence intervals (CI). The *I* ^2^ test was calculated as a quantitative measure of heterogeneity, with *I* ^2^ ≥ 50% considered as substantial heterogeneity (Yatabe et al., 2018). The random effects model was used when *I* ^2^ was >50%; otherwise, the fixed effect model was used. Ranking probabilities were calculated by the surface under the cumulative ranking analysis (SUCRA) for each outcome to increase the estimated precision of the effect sizes. The larger the SUCRA value, the better the LMWHs (Zhou et al., 2018). Considering the difference of patients involved, we further performed subgroup analysis based on patient characteristics (patients with DVT, patients with cardiac disease, medical patients, and patients >65 years old, > 70 years old, > 75 years old) and study characteristics (sample size >100, follow-up shorter than 60 days, follow-up longer than 60 days, single-center, multicenter, RCTs before 2010, and RCTs after 2010).

## Results

### Study Characteristics

**FIGURE 1:**
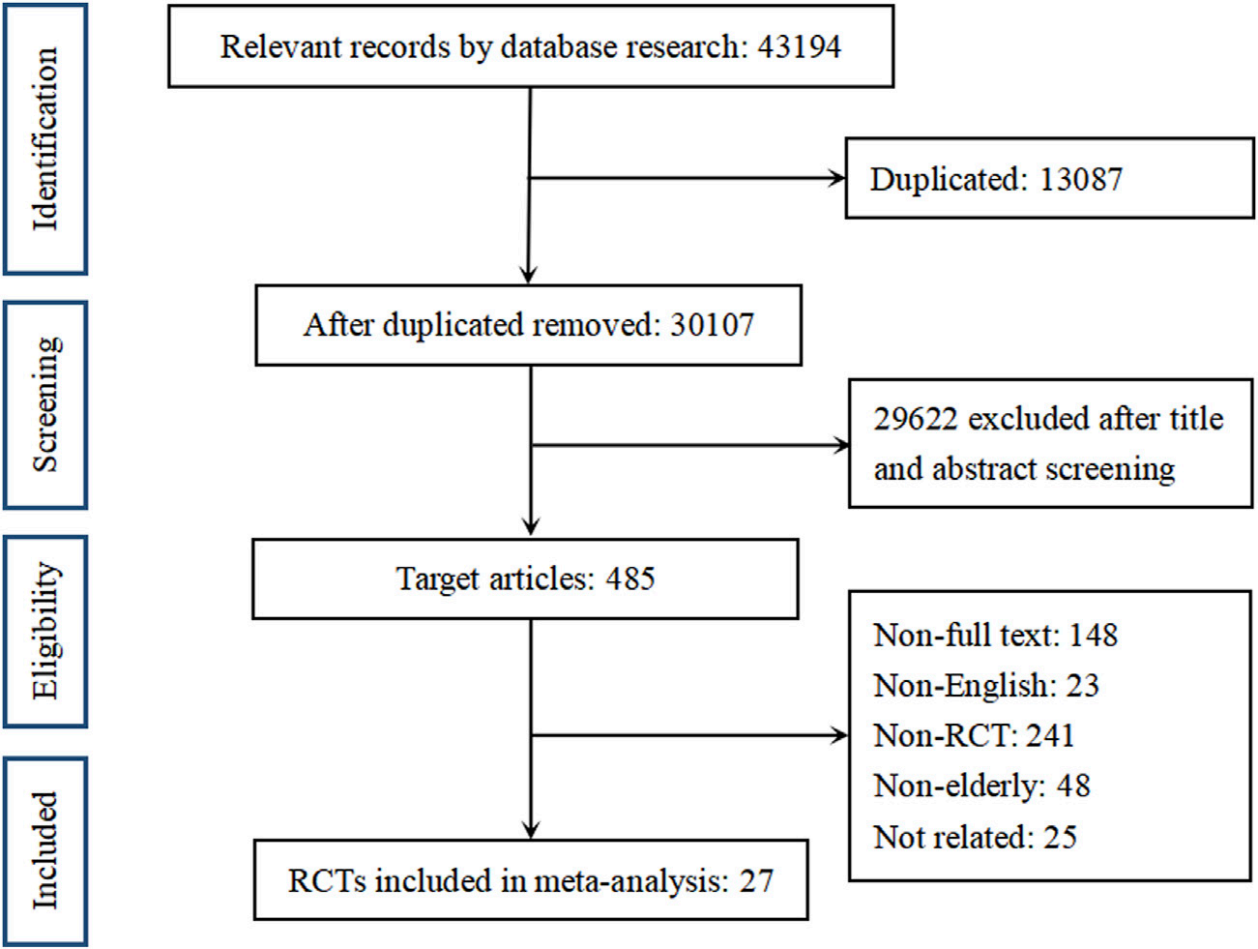
Flow diagram of study selection. RCT, randomized controlled trial.

We finally included 27 RCTs in our network meta-analysis. The flow of our study selection is shown in Figure 1. A total of 30,441 patients were included, including 13,351 in the LMWHs group and 17,090 in the control group.

**FIGURE 2:**
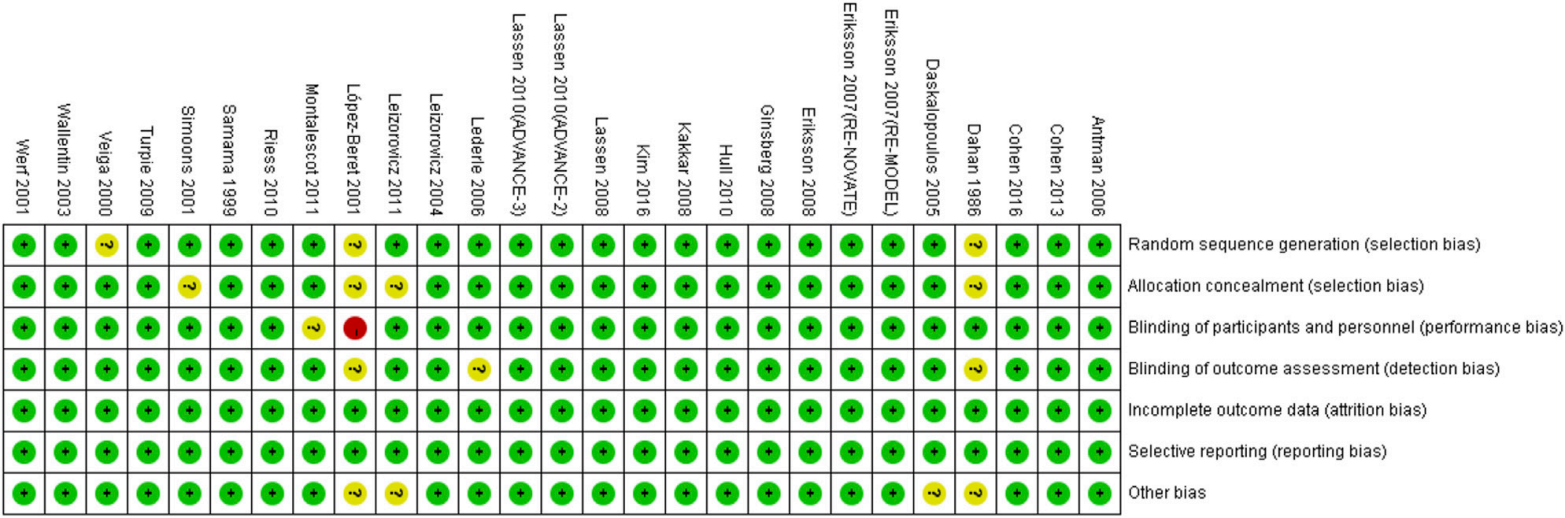
Risk bias of the included RCTs. + (green), low risk; −(red), high risk; ? (yellow), unclear risk.

The characteristics of included RCTs are shown in Supplementary Table S4. The LMWHs included certoparin (*n* = 1), enoxaparin (*n* = 21), tinzaparin (*n* = 2), nadroparin (*n* = 1), and dalteparin (*n* = 2). The population included DVT (*n* = 4), cardiac disease (*n* = 6), medical (*n* = 7), and orthopedic surgery (*n* = 10) patients. All subjects were 60 years or older. The data were from five original RCTs (Dahan et al., 1986; Veiga et al., 2000; Lederle et al., 2006; Riess et al., 2010; Leizorovicz et al., 2011), 10 subgroup analyses of 10 main RCTs (Samama et al., 1999; Lopez-Beret et al., 2001; Simoons, 2001; Leizorovicz et al., 2004; Daskalopoulos et al., 2005; Antman et al., 2006; Hull et al., 2010; Montalescot et al., 2011; Cohen et al., 2016; Kim et al., 2016), one meta-analysis (Sardar et al., 2014), and four pooled analyses of RCTs (Sinnaeve et al., 2006; Friedman et al., 2010; Turpie et al., 2011; Pineo et al., 2013). The risk bias of included RCTs is shown in Figure 2.

There was no direct comparison among different LMWHs. When compared with other treatments, LMWHs were definitely more effective than placebo in preventing VTE or VTE-related death (RR 0.36, 95% CI 0.25–0.53) and safer than acenocoumarol regarding risk of bleeding events (RR 0.67, 95% CI 0.49–0.90). However, LMWHs were less effective than novel oral anticoagulant (NOACs) in preventing VTE or VTE-related death (RR 1.59, 95 %CI 1.33–1.91). LMWHs show efficacy and safety similar to that of UFHs (Supplementary Table S6).

### Efficacy

**FIGURE 3:**
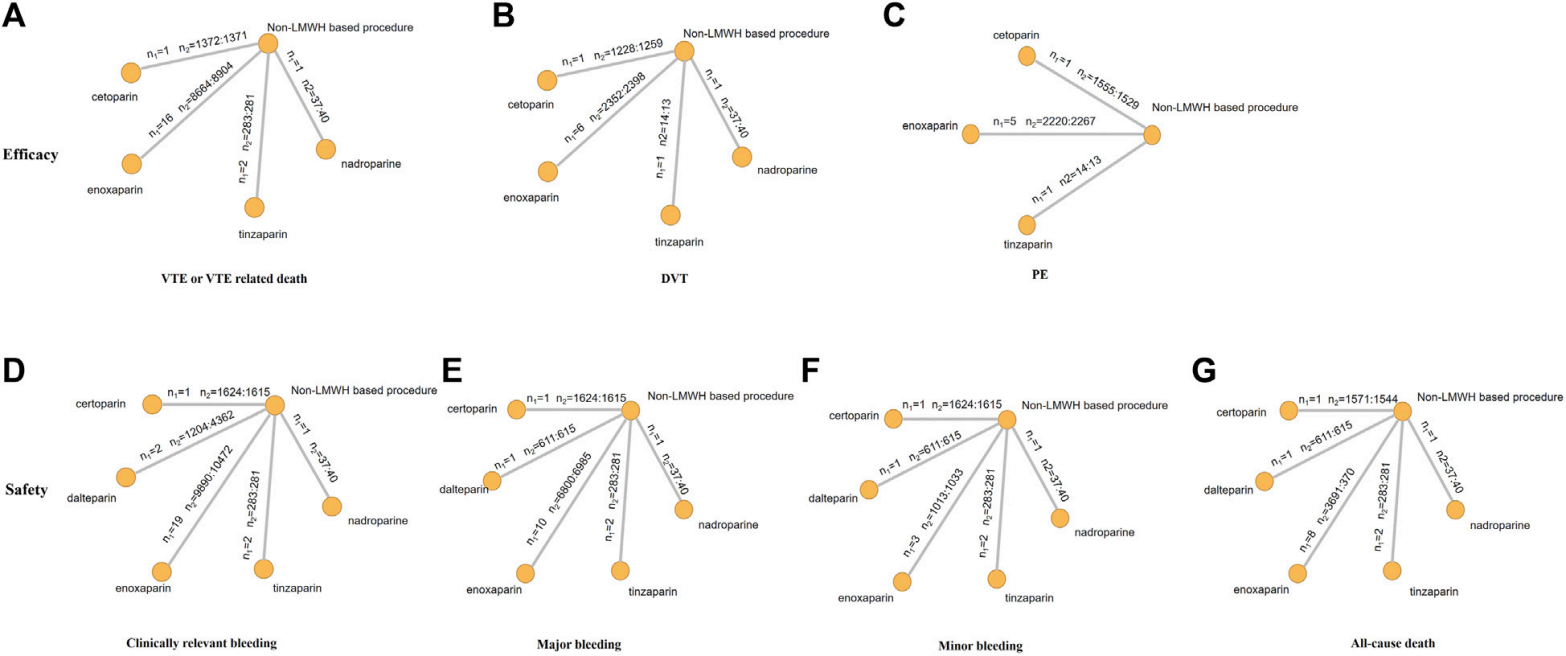
Network structure for all outcomes. The network plots show direct comparison of different treatments. *n*1, number of RCTs; *n*2, number of patients (LMWHs group vs control group).

**FIGURE 4:**
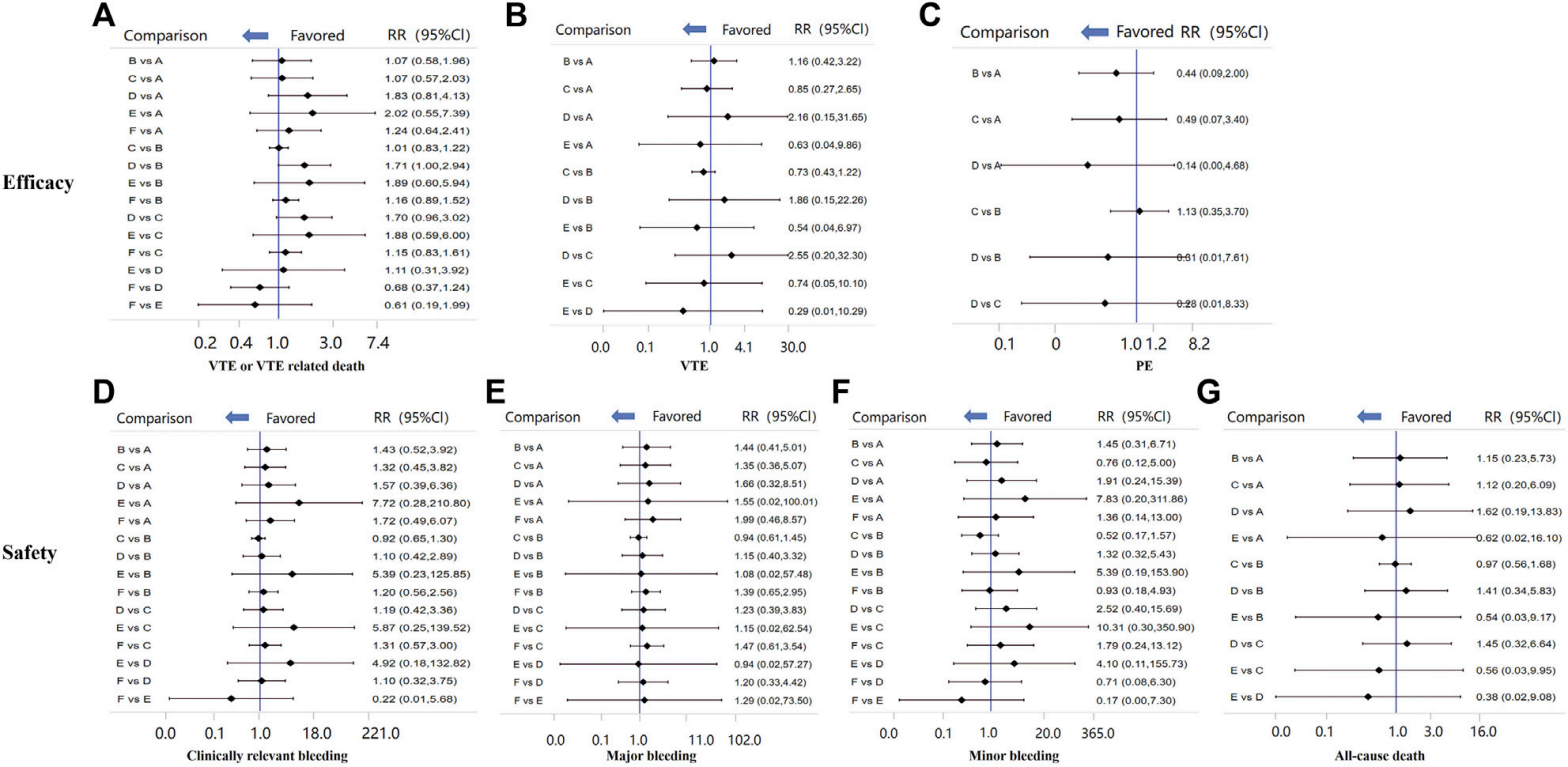
Comparisons of all efficacy and safety outcomes among different LMWHs. RR, relative risk; 95%CI, 95% confidence interval. VTE, venous thromboembolism; DVT, deep vein thrombosis.

**FIGURE 5:**
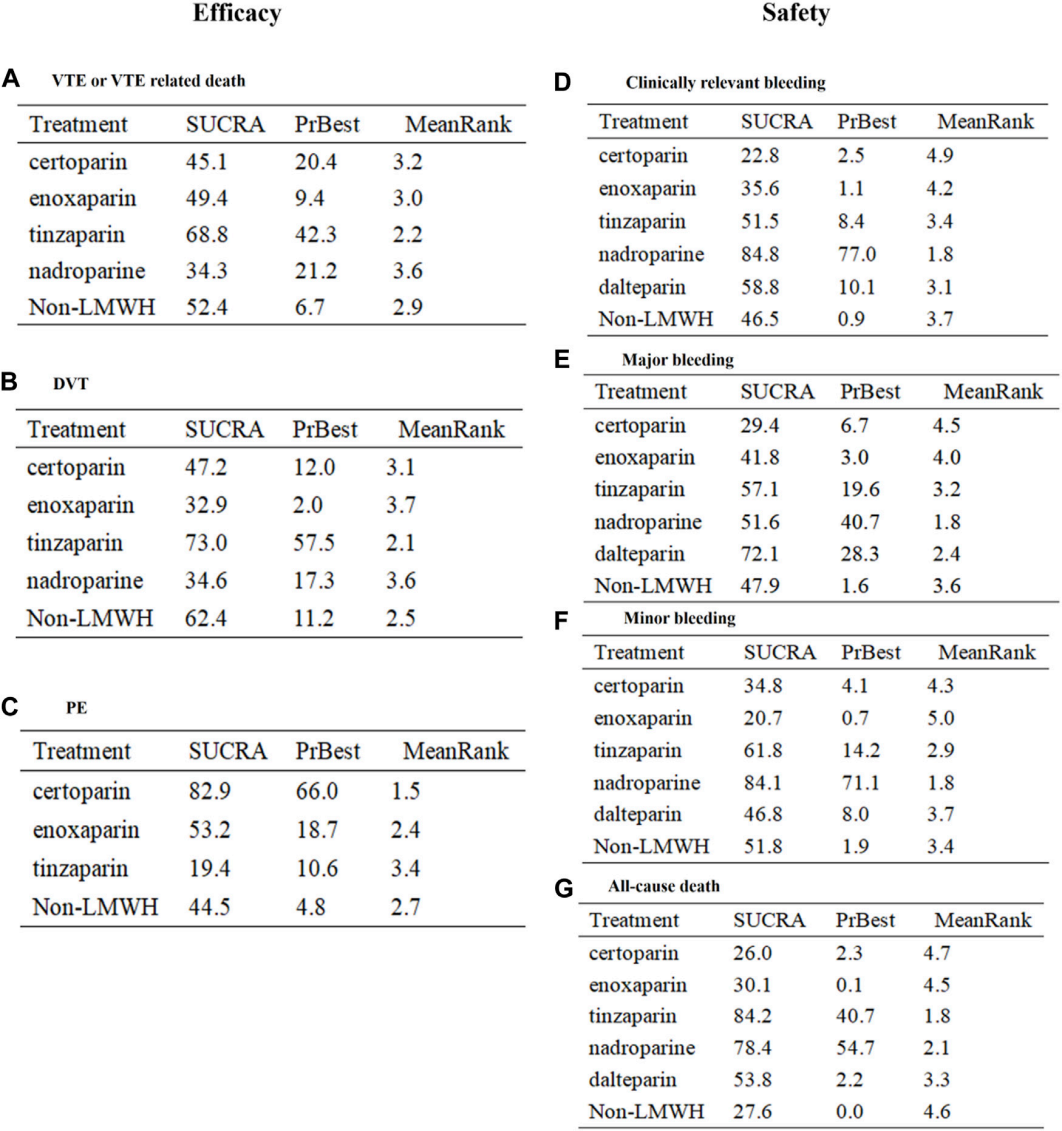
SUCRA of LMWHs for all efficacy and safety outcomes. RR, relative risk; 95% CI, 95% confidence interval. VTE, venous thromboembolism; DVT, deep vein thrombosis.

The endpoint of VTE or VTE-related death was reported in 20 RCTs and involved four types of LMWH (certoparin, *n* = 1; enoxaparin, *n* = 16; nadroparin, *n* = 1; tinzaparin, *n* = 2) (Figure 3A). As shown in Figure 3B, the four kinds of LMWHs show no significant difference in preventing incidence of VTE or VTE-related death (Figure 4A). The rank probabilities of the four LMWHs are shown in Figure 5A, whereby tinzaparin provided the most benefit in VTE or VTE-related death (SUCRA 68.8%, cumulative probability 42.3%), whereas nadroparin provided the least benefit (SUCRA 34.3%, cumulative probability 21.2%).

The indirect analysis of DVT is shown in Figure 3B, which included nine RCTs and involved four types of LMWH (certoparin, *n* = 1; enoxaparin, *n* = 6; nadroparin, *n* = 1; tinzaparin, *n* = 1). There was no significant difference in the incidence of different LMWHs (Figure 4B). Tinzaparin performed best with regard to efficacy in VTE (SUCRA 73.0%, cumulative probability 57.5%), and enoxaparin had the worst efficacy (SUCRA 32.9%, cumulative probability 2.0%) (Figure 5B).

PE was reported in seven RCTs and included three LMWHs (certoparin, *n* = 1; tinzaparin, *n* = 1; enoxaparin, *n* = 5) (Figure 3C). The three LMWHs showed no significant difference regarding PE (Figure 4C). Certoparin was ranked first (SUCRA 82.9%, cumulative probability 66.0%) and tinzaparin was ranked last (SUCRA 19.4%, cumulative probability 10.6%) (Figure 5C).

### Safety

Clinically relevant bleeding included major and minor bleeding. The results of indirect analysis are shown in Figure 3D, including 25 RCTs and five kinds of LMWHs (enoxaparin, *n* = 19; tinzaparin, *n* = 2; nadroparin, *n* = 1, dalteparin, *n* = 2; certoparin, *n* = 1). The five kinds of LMWH showed no significant difference in the incidence of clinically relevant bleeding (Figure 4D). The rank probabilities indicated that nadroparin had a 77.0% probability of being the best therapy (SUCRA: 84.8%) to prevent incidence of clinically relevant bleeding, whereas certoparin was the worst therapy (SUCRA: 22.8%, cumulative probability: 2.5%) (Figure 5D).

Incidence of major bleeding was reported in 15 RCTs (one was excluded because no incidence of major bleeding was observed) and included five LMWHs (Figure 3E). Eight RCTs reported incidence of minor bleeding with five LMWHs involved (Figure 3F). The indirect comparisons of the two endpoints are shown in Figures 4E,F. Different LMWHs showed no significant difference in both major and minor bleeding. Dalteparin showed the best efficacy for major bleeding (SUCRA 72.1%, cumulative probability 28.3%), and certoparin showed the worst efficacy (SUCRA 29.4%, cumulative probability 6.7%) (Figure 5E). Nadroparin had a 71.1% probability of being the best therapy to prevent the incidence of minor bleeding (SUCRA 84.1%), whereas enoxaparin was ranked last (SUCRA 20.7%, cumulative probability 0.7%) (Figure 5F).

A total of 14 RCTs reported the incidence of all-cause death after treatment with anticoagulants with five LMWHs involved (enoxaparin, *n* = 8; tinzaparin, *n* = 2; nadroparin, *n* = 1; dalteparin, *n* = 1; certoparin, *n* = 1; one was excluded because no incidence of major bleeding was observed) (Figure 3G). Indirect comparison indicated that no LMWHs showed significant superiority regarding the risk of all-cause death (Figure 4G). The rank probabilities indicated that tinzaparin delivered the greatest benefit for the risk of all-cause mortality (SUCRA 84.2%, cumulative probability 40.7%), and certoparin provided the least benefit (SUCRA 26.0%, cumulative probability 2.3%) (Figure 5G).

### Publication Bias

For comparisons involving 10 or more studies, Begg funnel plots for studies of VTE or VTE-related death, clinically relevant bleeding, major bleeding, and all-cause death were symmetrical (Supplementary Figure S1). Thus, there was no statistically significant evidence of publication bias in the studies reported herein.

### Subgroup Analysis

To verify the stability of our results, we further performed subgroup analyses in populations with different characteristics: patients with DVT (Supplementary Table S7), internal medical patients (Supplementary Table S8), patients with cardiac disease (Supplementary Table S9) (orthopedic surgery patients were excluded because they used fewer than two types of LMWH), patients >65 years old (Supplementary Table S10), patients >70 years old (Supplementary Table S11), and patients >75 years old (Supplementary Table S12); and studies with different characteristics: sample size >100 (Supplementary Table S13), follow-up time <60 days (Supplementary Table S14), follow-up time >60 days (Supplementary Table S15), multicenter (Supplementary Table S16), single-center (Supplementary Table S17), RCTs before 2010 (Supplementary Table S18), and RCTs after 2010 (Supplementary Table S19). Different LMWHs showed similar efficacy and safety with regard to the incidence of VTE or VTE-related death, clinically relevant bleeding, and all-cause mortality. In addition, tinzaparin showed the greatest overall benefit in VTE and VTE-related death and all-cause mortality in our ranking analysis, whereas nadroparin was ranked first in reducing the incidence of bleeding events.

Considering that enoxaparin constituted the majority of LMWHs we included, we conducted a subgroup analysis that excluded the RCTs of enoxaparin. After these 21 RCTs were discarded, dalteparin was associated with a decreased risk of clinically relevant bleeding (RR 1.76, 95% CI 1.10–2.82) in comparison with cetoparin (Supplementary Table S20).

## Discussion

To our knowledge, this is the first study that compares the efficacy and safety of different LMWHs in the elderly. A total of 27 RCTs and 30,441 elderly subjects were included in our network analysis with five LMWHs included in the intervention group and other treatments (placebo, UFH, acenocoumarol, NOACs) in the control group. In the head-to-head comparisons, LMWHs were less effective than NOACs but more effective than placebo, safer than acenocoumarol, and similar to UFH in terms of efficacy and safety. In the network analysis, no significant difference was observed in all efficacy (VTE or VTE-related death, DVT, PE) and safety (clinically relevant bleeding, major bleeding, minor bleeding) outcomes among the five LMWHs. In general, the SUCRA probabilities indicated that tinzaparin provided the most benefit in preventing VTE and VTE-related death and in decreasing the risk of death. Nadroparin ranked first in reducing the incidence of bleeding events. The results were also supported by our subgroup analyses and promise to provide a reference for clinicians when making clinical decisions.

Given their susceptibility to various diseases, multiple prescriptions, and attenuation of renal and liver function, the efficacy and safety of anticoagulants are both important to the elderly. A meta-analysis including 29,403 elective postarthroplasty elderly indicated that, with NOACs, the risk of VTE or VTE-related death was similar to that with LMWHs (odds ratio [OR] 0.62, 95% CI 0.30–1.26; *p* = 0.18; *I* ^*2*^ = 44%), but the bleeding risk was significantly lower (OR 0.71, 95% CI 0.53–0.94; *p* = 0.02; *I* ^*2*^ = 0%) (Pathak et al., 2015). However, our direct comparisons showed that LMHWs are less effective than NOACs although the safety profile showed no significant difference. In addition to the elderly after elective postarthroplasty, we also included older patients with other disease status, which may contribute to the controversy over the efficacy and safety of LMWHs and NOACs. LMWHs showed efficacy and safety effects equivalent to those of UFH in our analysis, and the results were supported by two previous meta-analyses (Macki et al., 2019; Lin et al., 2021). In addition, LMWHs in our analysis were more effective than placebo and safer than acenocoumarol.

For reasons of better safety and efficacy, many guidelines recommend LMWHs for the treatment and prevention of VTE (Rhodes et al., 2017; Farge et al., 2019) as well as for the elderly during the perioperative period (Kozek-Langenecker et al., 2018). Furthermore, since the outbreak of COVID-19, LMWH has been widely used for anticoagulation in older COVID-19 patients (Kreidieh and Temraz, 2021). In patients with proximal DVT and PE, LMWHs are preferred over oral anticoagulants and UFH (Hozayen et al., 2021). In-hospital COVID-19 patients who were on anticoagulation with LMWHs or UFH had a significantly reduced risk of mortality (hazard ratio 2.26, 95% CI 1.17–4.37). A retrospective study by Pasquale et al. (Paolisso et al., 2020) based on 450 COVID-19 patients (mean age 67 years) showed that this protective effect was also associated with the dose of LMWH: compared with standard prophylactic doses (40–60 mg daily), moderate doses (40–60 mg twice daily) were significantly associated with lower in-hospital mortality (18.8 vs. 5.8%, *p* = 0.02). This conclusion was also supported by an RCT conducted by Spyropoulos (Spyropoulos et al., 2021). Compared with NOACs, LMWH also showed better benefit in reducing mortality, improving markers of cell death, and curtailing viral persistence (Pereyra et al., 2021).

Although no specific LMWH is recommended, and they all differ in their manufacturing processes and molecular weights as well as anti-Xa, antithrombin, and anticoagulant activities, the LMWHs reported herein did not show any difference in all efficacy and safety endpoints throughout the network analysis. Research on the pharmacokinetics of LMWH shows that LMWHs accumulated significantly only in patients with poor renal function (Stiekema et al., 1989; Rico et al., 2014). Moreover, a study by Siguret et al. on the cumulative effects of tinzaparin in the elderly showed no progressive increase in anti-Xa or anti-IIa activity after repeated administration for 10 days (Siguret et al., 2000). The authors conclude that there was no need to adjust the dosage of tinzaparin even in elderly individuals with renal insufficiency. This further verified the preferable safety of LMWHs in the elderly, and the deterioration of renal function did not lead to significant influence on their efficacy or safety. Furthermore, a study compared the efficacy of enoxaparin and dalteparin in patients with traumatic injury (Miano et al., 2018). Despite enoxaparin providing 30%–100% greater factor Xa inhibition than dalteparin, the 10-year real-world study indicated no significant difference in efficacy between enoxaparin and dalteparin. Thus, the antithrombotic effect of LMWHs may be mediated by mechanisms other than anti-Xa (Frydman, 1996).

As reflected in our included RCTs, enoxaparin is the most widely used LMWH in clinical practice. A review indicates that, of enoxaparin, dalteparin, and nadroparin, only enoxaparin had sustained clinical and economic benefits compared with UFH in patients with unstable angina/non-ST-segment elevation myocardial infarction (Cohen, 2003). Therefore, different LMWHs may differ in their efficacy and safety, and enoxaparin did not show any advantages in our included elderly population. This may largely be because of the imbalance in the number of RCTs of different LMWHs with studies on other LMWHs besides enoxaparin being inadequate. After the RCTs of enoxaparin were removed from our analysis, dalteparin showed a lower bleeding risk than cetoparin. Thus, to eliminate the bias caused by the imbalance in the number of patients and increase the reliability of our results, more studies are needed to explore the efficacy and safety of other LMWHs in the elderly.

Tinzaparin, as the largest LMWH (molecular weight 6500 Da) in the clinic, is produced by the enzymatic degradation of porcine-derived UFH (Ageno et al., 2019) and is currently recommended as the first-line treatment for cancer-associated thrombosis (Ageno et al., 2019). In the prevention of DVT and/or PE, the efficacy of subcutaneous injection of tinzaparin in orthopedic surgery patients was superior to oral warfarin (Hoy et al., 2010). Moreover, our SUCRA probabilities suggest that tinzaparin performed best in preventing VTE and VTE-related and all-cause death. Nadroparin is an LMWH with a mean molecular weight of 4500 Da that is used for the treatment and prevention of long-term thromboembolism disorders (Barradell and Buckley, 1992). As early as 1997, a review summarized that nadroparin was more effective and safer than UFH in older patients (Barradell and Buckley, 1992). Our SUCRA probabilities showed that nadroparin performed best in decreasing the risk of bleeding. A recent pilot study indicates that nadroparin, in comparison with dabigatran or rivaroxaban, showed no difference in bleeding complications in patients with total knee arthroplasty surgery (van der Veen et al., 2021). A retrospective study compared the safety and efficacy of tinzaparin and nadroparin in neurosurgery and showed no significant difference in bleeding events and incidence of VTE (Wilhelmy et al., 2021). Thus, the superiority of tinzaparin and nadroparin in the elderly needs to be further studied.

We concede that there are some limitations to our analysis. Because no studies directly compare the efficacy and safety of different LMWHs in the elderly, our network meta-analysis only includes indirect comparative evidence and lacks direct evidence. We include data from some RCTs, existing meta-analyses, and pooled analyses. Therefore, the details of patients (such as average age, gender, BMI, and so on) were not clear. The RCTs included in our study have some differences in the participating population, treatment protocols, and definition of outcomes. There was a high degree of statistical heterogeneity at some endpoints.

## Conclusion

In the elderly, LMWH is a safer anticoagulant in comparison with other agents. Among different LMWHs, no significant difference in the incidence of VTE or VTE-related death, DVT, PE, clinically relevant bleeding, major bleeding, minor bleeding, or all-cause death was observed in our network comparison. The rank probability analysis indicated that nadroparin seems to be safer for elderly patients at high risk of bleeding, and tinzaparin is more effective for those with a low bleeding risk. Certainly, the results need to be further verified in more rigorous clinical research.

## Data Availability

The original contributions presented in the study are included in the article/Supplementary Material, further inquiries can be directed to the corresponding authors.
