# Supplementary material for: Safety and Efficacy of Low Molecular Weight Heparin for Thromboprophylaxis in the Elderly: A Network Meta-Analysis of Randomized Clinical Trials
Source: Front Pharmacol. 2021 Dec 10;12:783104. doi: 10.3389/fphar.2021.783104 (PMC8703065; doi:10.3389/fphar.2021.783104)
Supplement: Supplementary file 1 [file DataSheet1.docx]

Supplementary Material

1. **Supplementary Figures and Tables**

**1.1 Supplementary Figure**


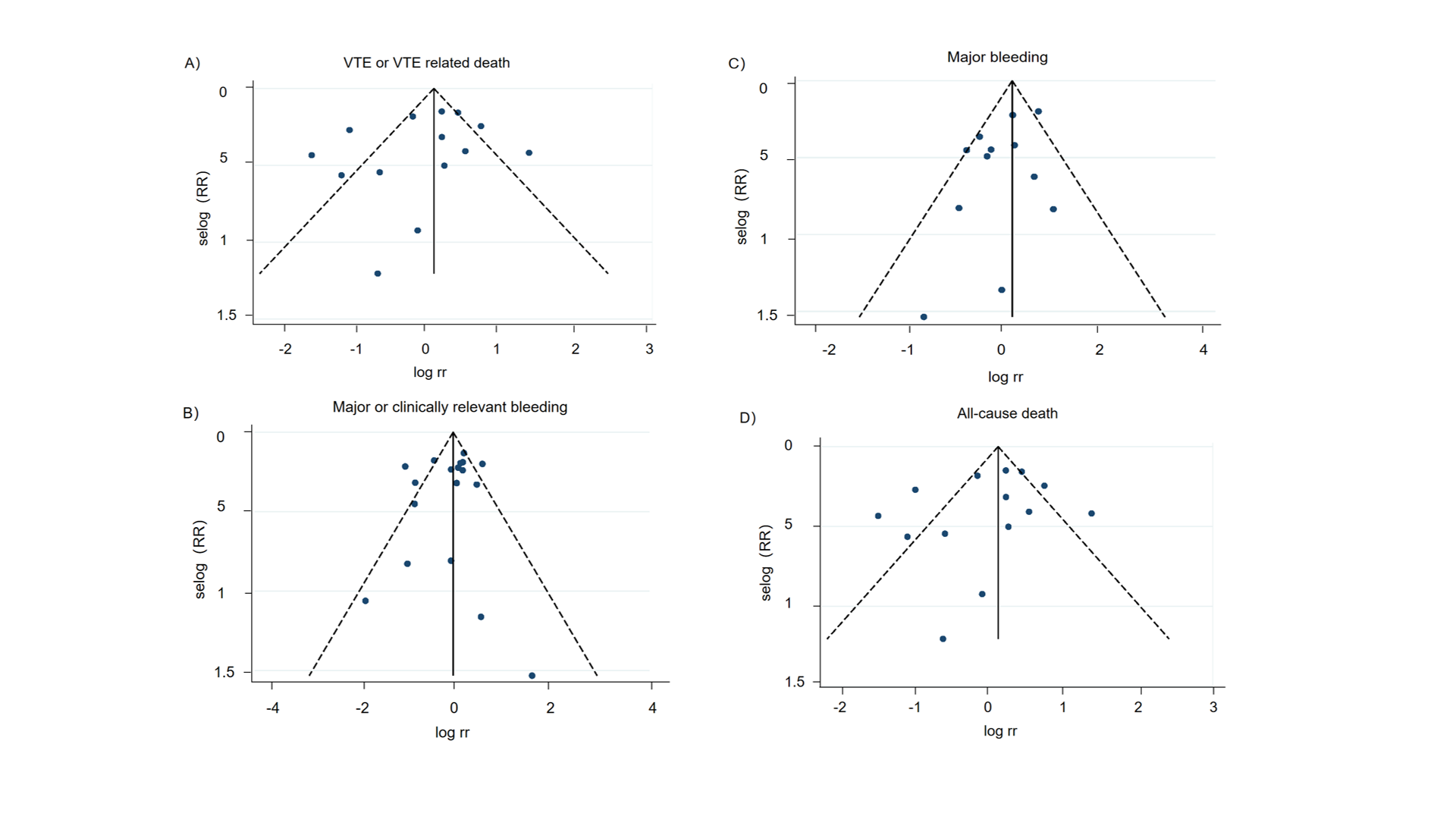


**Supplementary Figure 1.** Funnel plots for publication bias.The dotted lines show 95% confidence intervals around the overall summary estimate calculated using a random effect model.

**1.2 Supplementary Tables**

**Table S1.** **Search strategy in pubmed. Previous -** **July 22, 2020.**

| #1 | "Heparin, Low-Molecular-Weight"[Mesh] | 12500 |
| --- | --- | --- |
| #2 | "heparin*"[Title/Abstract] OR "LMWH"[Title/Abstract] OR "nadroparin*"[Title/Abstract] OR "fraxiparin*"[Title/Abstract] OR "enoxaparin"[Title/Abstract] OR "Clexane"[Title/Abstract] OR "klexane"[Title/Abstract] OR "lovenox"[Title/Abstract] OR "dalteparin"[Title/Abstract] OR "Fragmin"[Title/Abstract] OR "ardeparin"[Title/Abstract] OR "normiflo"[Title/Abstract] OR "tinzaparin"[Title/Abstract] OR "logiparin"[Title/Abstract] OR "Innohep"[Title/Abstract] OR "certoparin"[Title/Abstract] OR "sandoparin"[Title/Abstract] OR "reviparin"[Title/Abstract] OR "clivarin*"[Title/Abstract] OR "danaproid"[Title/Abstract] OR "danaparoid"[Title/Abstract] OR "bemiparin"[Title/Abstract] OR "Troparin"[Title/Abstract] | 89655 |
| #3 | (((((((((((((("antixarin"[Title/Abstract] OR "ardeparin*"[Title/Abstract]) OR "bemiparin*"[Title/Abstract]) OR "Zibor"[Title/Abstract]) OR "cy 222"[Title/Abstract]) OR "embolex"[Title/Abstract]) OR "monoembolex"[Title/Abstract]) OR "Mono-embolex"[Title/Abstract]) OR "parnaparin*"[Title/Abstract]) OR "tedelparin"[Title/Abstract]) OR "Kabi-2165"[Title/Abstract]) OR ((("dalteparin"[MeSH Terms] OR "dalteparin"[All Fields]) OR "Kabi-2165"[Title/Abstract]))) OR "emt-966"[Title/Abstract]) OR "emt-967"[Title/Abstract]) OR "pk-10169"[Title/Abstract]) OR "pk10169"[Title/Abstract] | 1631 |
| #4 | "cy-216"[Title/Abstract] OR "cy216"[Title/Abstract] OR "seleparin*"[Title/Abstract] OR "seleparin*"[Title/Abstract] OR "tedelparin"[Title/Abstract] OR "Liquemine"[Title/Abstract] | 105 |
| #5 | "fr-860"[Title/Abstract] | 12 |
| #6 | wy90493 or wy-90493 | 0 |
| #7 | "kb-101"[Title/Abstract] OR "kb101"[Title/Abstract] OR "lomoparan"[Title/Abstract] OR "orgaran"[Title/Abstract] | 107 |
| #8 | "parnaparin"[Title/Abstract] OR "fluxum"[Title/Abstract] OR "op 2123"[Title/Abstract] OR "parvoparin"[Title/Abstract] | 62 |
| #9 | "AVE5026"[Title/Abstract] OR "M118"[Title/Abstract] OR "RO-14"[Title/Abstract] | 75 |
| #10 | #1 OR #2 OR #3 OR #4 OR #5 OR #6 OR #7 OR #8 OR #9 | 91993 |
| #11 | "elder people"[Title/Abstract] OR elderly[Title/Abstract] OR "the aged"[Title/Abstract] OR aging[Title/Abstract] OR "old people"[Title/Abstract] OR elder[Title/Abstract] OR "old man"[Title/Abstract] OR "the old"[Title/Abstract] OR "the elderly"[Title/Abstract] | 579757 |
| #12 | aged[MeSH Terms] | 3117219 |
| #13 | #11 OR #12 | 3423838 |
| #14 | #10 AND #13 | 15225 |

**Table S2. Search strategy in embase. Previous - July 22, 2020.**

| #1 | 'low molecular weight heparin'/exp | 62879 |
| --- | --- | --- |
| #2 | heparin*:ab,ti OR lmwh:ab,ti OR nadroparin*:ab,ti OR fraxiparin*:ab,ti OR enoxaparin:ab,ti OR clexane:ab,ti OR klexane:ab,ti OR lovenox:ab,ti OR dalteparin:ab,ti OR fragmin:ab,ti OR ardeparin:ab,ti OR normiflo:ab,ti OR tinzaparin:ab,ti OR logiparin:ab,ti OR innohep:ab,ti OR certoparin:ab,ti OR sandoparin:ab,ti OR reviparin:ab,ti OR clivarin*:ab,ti OR danaproid:ab,ti OR danaparoid:ab,ti OR bemiparin:ab,ti OR bioparin:ab,ti OR alphaparin:ab,ti OR troparin:ab,ti | 123189 |
| #3 | antixarin:ab,ti OR ardeparin*:ab,ti OR bemiparin*:ab,ti OR zibor:ab,ti OR 'cy 222':ab,ti OR embolex:ab,ti OR monoembolex:ab,ti OR 'mono embolex':ab,ti OR parnaparin*:ab,ti OR 'rd 11885':ab,ti OR tedelparin:ab,ti OR 'kabi 2165':ab,ti OR 'emt 966':ab,ti OR 'emt 967':ab,ti OR 'pk 10169':ab,ti OR pk10169:ab,ti | 484 |
| #4 | cy 216':ab,ti OR cy216:ab,ti OR tedegliparin:ab,ti OR seleparin*:ab,ti OR tedegliparin*:ab,ti OR tedelparin:ab,ti OR liquemine:ab,ti | 120 |
| #5 | fr 860':ab,ti | 14 |
| #6 | 'wy 90493':ab,ti OR wy90493:ab,ti | 0 |
| #7 | 'kb 101':ab,ti OR kb101:ab,ti OR lomoparan:ab,ti OR orgaran:ab,ti | 162 |
| #8 | parnaparin:ab,ti OR fluxum:ab,ti OR lohepa:ab,ti OR lowhepa:ab,ti OR 'op 2123':ab,ti OR parvoparin:ab,ti | 96 |
| #9 | parvoparin:ab,ti OR m118:ab,ti OR 'ro 14':ab,ti | 76 |
| #10 | #1 OR #2 OR #3 OR #4 OR #5 OR #6 OR #7 OR #8 OR #9 | 156667 |
| #11 | 'aged'/exp | 3109454 |
| #12 | 'elder people':ab,ti OR elderly:ab,ti OR 'the aged':ab,ti OR aging:ab,ti OR 'old people':ab,ti OR elder:ab,ti OR 'old man':ab,ti OR 'the old':ab,ti OR 'the elderly':ab,ti | 745978 |
| #13 | #11 OR #12 | 3487084 |
| #14 | #10 AND #13 | 24131 |

**Table S3 . Search strategy in Cochrane. Previous - July 22, 2020.**

| #1 | MeSH descriptor: [Heparin, Low-Molecular-Weight] explode all trees | 1953 |
| --- | --- | --- |
| #2 | (heparin*):ti,ab,kw OR (LMWH):ti,ab,kw OR (nadroparin*):ti,ab,kw OR (fraxiparin*):ti,ab,kw OR (enoxaparin):ti,ab,kw | 13352 |
| #3 | (Clexane):ti,ab,kw OR (klexane):ti,ab,kw OR (lovenox):ti,ab,kw OR (dalteparin):ti,ab,kw OR (Fragmin):ti,ab,kw | 1036 |
| #4 | (ardeparin):ti,ab,kw OR (normiflo):ti,ab,kw OR (tinzaparin):ti,ab,kw OR (logiparin):ti,ab,kw OR (Innohep):ti,ab,kw | 278 |
| #5 | (certoparin):ti,ab,kw OR (sandoparin):ti,ab,kw OR (reviparin):ti,ab,kw OR (clivarin*):ti,ab,kw OR (danaproid):ti,ab,kw | 159 |
| #6 | (danaparoid):ti,ab,kw OR (bemiparin):ti,ab,kw OR (Troparin):ti,ab,kw OR (antixarin):ti,ab,kw OR (ardeparin*):ti,ab,kw | 136 |
| #7 | (bemiparin*):ti,ab,kw OR (Zibor):ti,ab,kw OR (cy 222):ti,ab,kw OR (embolex):ti,ab,kw OR (monoembolex):ti,ab,kw | 120 |
| #8 | (Mono-embolex):ti,ab,kw OR (parnaparin*):ti,ab,kw OR (tedelparin):ti,ab,kw OR (Kabi-2165):ti,ab,kw OR (dalteparin):ti,ab,kw | 834 |
| #9 | (Kabi-2165):ti,ab,kw OR (emt-966):ti,ab,kw OR (emt-967):ti,ab,kw OR (pk-10169):ti,ab,kw OR (pk10169):ti,ab,kw | 45 |
| #10 | (cy-216):ti,ab,kw OR (cy216):ti,ab,kw OR (seleparin*):ti,ab,kw OR (tedelparin):ti,ab,kw OR (Liquemine):ti,ab,kw (Word variations have been searched) | 667 |
| #11 | (fr-860):ti,ab,kw OR (wy90493):ti,ab,kw OR (wy-90493):ti,ab,kw OR (kb-101):ti,ab,kw OR (kb101):ti,ab,kw | 8 |
| #12 | (lomoparan):ti,ab,kw OR (orgaran):ti,ab,kw OR (parnaparin):ti,ab,kw OR (fluxum):ti,ab,kw OR (op 2123):ti,ab,kw | 83 |
| #13 | (parvoparin):ti,ab,kw OR (AVE5026):ti,ab,kw OR (M118):ti,ab,kw OR (fluxum):ti,ab,kw AND (low molecular weight heparin):ti,ab,kw | 34 |
| #14 | #1 OR #2 OR #3 OR #4 OR #5 OR #6 OR #7 OR #8 OR #9 OR #10 OR #11 OR #12 OR #13 | 13700 |
| #15 | MeSH descriptor: [Aged] in all MeSH products | 203287 |
| #16 | (“elder people” OR elderly OR “the aged” OR aging OR “old people” OR elder OR “old man” OR “the old” OR “the elderly”):ti,ab,kw | 99359 |
| #17 | #15 OR #16 | 277827 |
| #18 | #14 AND #17 | 3841 |

**Table S4. Characteristic of the included studies.**

| Author, year | No. of sites | Patients | Definition of the elderly | Number of patients | | Treatment | | Age | | Female | | Weight | |
| --- | --- | --- | --- | --- | --- | --- | --- | --- | --- | --- | --- | --- | --- |
|  |  |  |  | Intervention | Control | Intervention | Control | Intervention | Control | Intervention | Control | Intervention | Control |
| Riess 2010 | 172 | Acute medical illness | ≥70 | 1624 | 1615 | certoparin | UFH | 79.0±6.2 | 78.7±6.3 | 955 | 960 | 72.3±16.2 | 71.9±15.3 |
| Montalescot 2011 | 64 | Primary PCI for STEMI | ≥75 | 85 | 80 | enoxaparin | UFH | 80 | 82 | 37 | 41 | 67.5 | 69.5 |
| Hull 2010 | 370 | Acute medical illness | >75 | 878 | 903 | enoxaparin | placebo | 81.6 ± 4.4 | 81.4±4.3 | 493 | 549 | N/A | N/A |
| Kim 2016 | 1 | Elective primary THA | ≥60 | 167 | 166 | enoxaparin | rivaroxaban | 69.4 ± 6.7 | 68.6±6.4 | 107 | 102 | N/A | N/A |
| Cohen 2016 | 460 | Acute medical illness | ≥80 | 1363 | 1446 | enoxaparin | betrixaban | 84.2±3.5 | 84.4±3.7 | 827 | 870 | 74.7±15.1 | 74.0±15.1 |
| Veiga 2000 | 1 | Symptomatic proximal DVT | >75 | 50 | 50 | enoxaparin | acenocoumarol | 80.9 | 79.6 | 33 | 26 | 59.2 | 60.9 |
| Antman 2006 | 674 | STEMI | ≥75 | 1241 | 1291 | enoxaparin | UFH | N/A | N/A | N/A | N/A | N/A | N/A |
| Leizorovicz 2011 | 109 | Renally impaired with acute symptomatic lower limb DVT | ≥70 | 269 | 268 | tinzaparin | UFH | 82.9±5.7 | 82.6±5.8 | 177 | 166 | N/A | N/A |
| Lederle 2006 | 5 | Hospitalized general medical | ≥60 | 140 | 140 | enoxaparin | placebo | 71.3 | 72.1 | 1 | 3 | 85.1 | 85.5 |
| Dahan 1986 | 1 | Medical in-patients | ≥65 | 132 | 131 | enoxaparin | placebo | 79.9±6.8 | 80.1±6.9 | 51 | 85 | N/A | N/A |
| Samama 1999 | 60 | Acutely ill, immobilized, general medical | >75 | 147 | 157 | enoxaparin | placebo | N/A | N/A | N/A | N/A | N/A | N/A |
| López-Beret 2001 | 1 | Symptomatic DVT | >70 | 40 | 37 | Nadroparine | acenocoumarol | N/A | N/A | N/A | N/A | N/A | N/A |
| Daskalopoulos 2005 | 1 | Acute leg DVT | ≥71 | 14 | 13 | tinzaparin | UFH | N/A | N/A | N/A | N/A | N/A | N/A |
| Leizorovicz 2004 | 219 | Acutely ill medical | ≥75 | 611 | 615 | dalteparin | placebo | N/A | N/A | N/A | N/A | N/A | N/A |
| Cohen 2013 | 556 | Acute medical illness | ≥75 | 1565 | 1551 | enoxaparin | rivaroxaban | N/A | N/A | N/A | N/A | N/A | N/A |
| Simoons 2001 | 458 | ACS | ≥65 | 592 | 3747 | dalteparin | UFH | N/A | N/A | N/A | N/A | N/A | N/A |
| Werf 2001 | 575 | STEMI | >65 | 1102 | 1132 | enoxaparin | UFH | N/A | N/A | N/A | N/A | N/A | N/A |
| Wallentin 2003 | 88 | STEMI |  |  |  |  |  |  |  |  |  |  |  |
| Eriksson 2008 | 27 countries | TKA | ≥75 | 914 | 914 | enoxaparin | rivaroxaban | N/A | N/A | N/A | N/A | N/A | N/A |
| Kakkar 2008 | 123 | TKR |  |  |  |  |  |  |  |  |  |  |  |
| Lassen 2008 | 147 | TKR |  |  |  |  |  |  |  |  |  |  |  |
| Turpie 2009 | 131 | TKR |  |  |  |  |  |  |  |  |  |  |  |
| Eriksson 2007(RE-NOVATE) | 115 | TKR | ≥75 | 461 | 877 | enoxaparin | dabigatran | N/A | N/A | N/A | N/A | N/A | N/A |
| Ginsberg 2008 | 58 | TKR |  |  |  |  |  |  |  |  |  |  |  |
| Eriksson 2007(RE-MODEL) | 105 | TKR |  |  |  |  |  |  |  |  |  |  |  |
| Lassen 2010(ADVANCE-2) | 125 | TKR or same-day bilateral knee replacement | ≥65 | 1956 | 1957 | enoxaparin | apixaban | N/A | N/A | N/A | N/A | N/A | N/A |
| Lassen 2010(ADVANCE-3) | 160 | TKR or revision of a previously inserted hip prosthesis |  |  |  |  |  |  |  |  |  |  |  |

STEMI , ST-elevation myocardial infarction; DVT, deep vein thrombosis, THA, total hip arthroplasty; TKR, total knee replacement; PCI, percutaneous coronary intervention ; ACS, acute coronary syndromes

Table S5. Definition of the outcomes in original RCTs.

|  | **Bleeding** | **VTE** | **All-cuase death** |
| --- | --- | --- | --- |
| Riess 2010 | ISTH | Proximal DVT, symptomatic non-fatal PE and VTE-related death | All-cuase death |
| Montalescot 2011 | STEEPLE | N/A | All-cuase death |
| Hull 2010 | ISTH | Asymptomatic DVT, symptomatic PE, or fatal PE | All-cuase death |
| Kim 2016 | More than six units of packed red blood cells | Asymptomatic DVT, symptomatic DVT, symptomatic PE, and death from all causes which cannot exclude VTE | Fatal PE or death |
| Cohen 2016 | ISTH | Asymptomatic proximal DVT, symptomatic proximal or distal DVT, symptomatic nonfatal PE, or death from VTE | VTE-related death |
| Veiga 2000 | ISTH | Recurrence of DVT or PE confirmed by imaging diagnosis or necropsy | All-cuase death |
| Antman 2006 | TIMI | N/A | N/A |
| Leizorovicz 2011 | Major bleeding:ISTH;  Minor bleedings: hematoma greater than 100 cm^2^ , epistaxis lasting more than 5 min or repetitive, macroscopic hematuria, gastrointestinal bleeds, pulmonary bleeds and any other bleed that resulted in clinical consequences for the patient. | Symptomatic recurrent VTE | N/A |
| Lederle 2006 | Major bleeding: fatal, life-threatening, potentially lifethreatening, or acute or subacute and led to reoperation or moderate or severe blood loss. | Symptomatic DVT; PE | All-cuase death |
| Dahan 1986 | N/A | DVT confirmed by imaging diagnosis | All-cuase death |
| Samama 1999 | N/A | DVT or PE confirmed by imaging diagnosis or necropsy | N/A |
| López-Beret 2001 | ISTH | DVT confirmed by  combining B-mode color imaging and Doppler scan measurements. | All-cuase death |
| Daskalopoulos 2005 | ISTH | DVT or PE confirmed by imaging diagnosis | All-cuase death |
| Leizorovicz 2004 | ISTH | N/A | All-cuase death |
| Cohen 2013 | ISTH | Asymptomatic proximal DVT, symptomatic proximal or distal DVT, symptomatic nonfatal PE, or death related to VTE | N/A |
| Simoons 2001 | TIMI | N/A | N/A |
| Werf 2001 | ICH | Symptomatic, recurrent DVT or PE confirmed by imaging diagnosis | N/A |
| Wallentin 2003 |  |  |  |
| Eriksson 2008 | ICH | Symptomatic VTE (symptomatic DVT and PE) and all-cause mortality | N/A |
| Kakkar 2008 |  |  |  |
| Lassen 2008 |  |  |  |
| Turpie 2009 |  |  |  |
| Eriksson 2007(RE-NOVATE) | Major bleeding: all bleeding events regardless of  the site of bleeding event | Major VTE and VTE-related mortality | N/A |
| Ginsberg 2008 |  |  |  |
| Eriksson 2007(RE-MODEL) |  |  |  |
| Lassen 2010(ADVANCE-2) | Major bleeding: ISTH;  Clinically relevant  non-major bleeding: acute clinically overt episodes  such as a wound hematoma, bruising/ecchymosis, gastrointestinal bleeding, hemoptysis, hematuria, or epistaxis | Symptomatic or asymptomatic proximal DVT  (popliteal, femoral or iliac vein thrombosis), non-fatal pulmonary embolism and VTE-related death | N/A |
| Lassen 2010(ADVANCE-3) |  |  |  |

ISTH：Major bleeding was defined as fatal bleeding, clinically overt bleeding associated with a fall in the hemoglobin concentration of more than 20 g L^-1^ as compared with the baseline hemoglobin concentration, clinically overt bleeding that required transfusion of two or more units of packed red cells or whole blood, or symptomatic bleeding in a critical area or organ (intracranial, intraspinal, retroperitoneal, and pericardial). Bleeding events that did not meet the abovementioned criteria were classified as minor.

STEEPLE：Major bleeding: Fatal bleeding; Retroperitoneal, intracranial, or intraocular bleeding; Bleeding that causes hemodynamic compromise requiring specific treatment; Bleeding that requires intervention (surgical or endoscopic) or decompression of a closed space to stop or control the event; Clinically overt bleeding, requiring any transfusion of ≥1 unit of packed red cells or whole blood; Clinically overt bleeding, causing a decrease in hemoglobin of ≥3 g/dl (or, if hemoglobin level not available, a decrease in hematocrit of ≥10%). Minor bleeding: Gross hematuria not associated with trauma (e.g., from instrumentation); Epistaxis that is prolonged, repeated, or requires plugging or intervention; Gastrointestinal hemorrhage; Hemoptysis; Subconjunctival hemorrhage;Hematoma >5 cm or leading to prolonged or new hospitalization; Clinically overt bleeding, causing a decrease in hemoglobin of 2 to 3 g/dl; Uncontrolled bleeding requiring protamine sulfate administration.

TIMI: Major bleeding during baseline hospital stay (for a maximum of 7 days) was defined as either intracranial haemorrhage or bleeding associated with a decrease in haemoglobin concentration of more than 50 g/L. Minor bleeding was defined as any of the following: spontaneous gross haematuria or haematemesis; observed blood loss with a decrease in haemoglobin concentration of more than 30 but less than or equal to 50 g/L; or decrease in haemoglobin concentration of more than 40 but less than or equal to 50 g/L without an identified bleeding site. Thrombocytopenia was defined as a platelet count of less than 50 000/L. All suspected occurrences of stroke or intracranial haemorrhage were adjudicated by an independent neurologist who was unaware of treatment assignment.

ICH: intracranial hemorrhage.**Table S6. Results of direct comparison between LMWHs and other anticoagulants**

| **Endpoints** | **Intervention vs control** | **RR** | **95%CI** | **I ^2^** |
| --- | --- | --- | --- | --- |
| **VTE or VTE related death** | **LMWHs vs placebo(n=4)** | **0.36** | **0.25,0.53** | 0.0% |
|  | LMWHs vs UFH(n=3) | 0.98 | 0.72,1.35 | 13.8% |
|  | **LMWHs vs NOACs(n=5)** | **1.59** | **1.33,1.91** | **58.8%** |
| **Clinically relevant bleeding** | LMWHs vs placebo(n=3) | 1.12 | 0.81,1.54 | 0.0% |
|  | LMWHs vs UFH(n=7) | 1.07 | 0.90,1.26 | 68.0% |
|  | **LMWHs vs acenocoumarol(n=2)** | **0.67** | **0.49,0.90** | **0.0%** |
|  | LMWHs vs NOACs(n=6) | 0.96 | 0.80,1.15 | 82.5% |
| **Death** | LMWHs vs placebo(n=4) | 1.09 | 0.87,1.37 | 0.0% |
|  | LMWHs vs UFH(n=5) | 1.06 | 0.87,1.29 | 35.0% |
|  | LMWHs vs acenocoumarol(n=2) | 0.76 | 0.47,1.22 | 26.1% |

RR, relative risk; 95%CI, 95% confidence interval.

LMWHs, low molecular heparins; NOACs: new oral anticoagulants; UFH, unfractionated heparin.

**Table S7. Subgroup analyses opatients with DVT**

| **Outcome** | **Comparison** | **RR（95%CI ）** | **SUCRA** |
| --- | --- | --- | --- |
| **VTE or VTE related death** | enoxaparin vs tinzaparin | 1.20(0.35,4.07) | enoxaparin:61.9 |
|  | enoxaparin vs nadroparine | 0.41(0.03,5.22) | **tinzaparin:77.2** |
|  | tinzaparin vs nadroparine | 0.34(0.03,4.01) | nadroparine:24.9 |
| **Clinically relevant bleeding** | enoxaparin vs tinzaparin | 6.13(0.73,51.47) | enoxaparin:4.5 |
|  | enoxaparin vs nadroparine | 32.37(0.84,1250.53) | tinzaparin:54.4 |
|  | tinzaparin vs nadroparine | 5.28(0.25,110.23) | **nadroparine:89.2** |
| **All-cause death** | enoxaparin vs tinzaparin | 1.20(0.43,3.37) | enoxaparin:49.6 |
|  | enoxaparin vs nadroparine | 1.32(0.31,5.62) | tinzaparin:68.5 |
|  | tinzaparin vs nadroparine | 1.11(0.31,3.92) | **nadroparine:68.9** |

RR, relative risk; 95%CI, 95% confidence interval.VTE, venous thrombus embolism; DVT, deep vein thrombosis.

**Table S8. Subgroup analyses of Medical patients**

| **Outcome** | **Comparison** | **RR（95%CI ）** | **SUCRA** |
| --- | --- | --- | --- |
| **VTE or VTE related death** | cetoparin vs enoxaparin | 0.76(0.13,3.72) | enoxaparin:21.6 |
|  |  |  | cetoparin:53.5 |
| **Clinically relevant bleeding** | cetoparin vs enoxaparin | 0.84(0.23,3.15) | enoxaparin:43.2 |
|  |  |  | cetoparin:23.6 |
| **Death** | cetoparin vs enoxaparin | 1.04(0.50,2.15) | enoxaparin:50.1 |
|  |  |  | cetoparin:43.2 |

RR, relative risk; 95%CI, 95% confidence interval.VTE, venous thrombus embolism; DVT, deep vein thrombosis.

**Table S9. Subgroup analyses of patients with [cardiac disease](C:/Users/%E5%88%98%E9%9D%92%E9%9D%92/AppData/Local/youdao/dict/Application/8.9.3.0/resultui/html/index.html#/javascript:;)**

| **Outcome** | **Comparison** | **RR（95%CI ）** | **SUCRA** |
| --- | --- | --- | --- |
| **Clinically relevant bleeding** | enoxaparin vs dalteparin | 1.13(0.41,3.12) | enoxaparin:49.2 |
|  |  |  | **dalteparin:63.3** |
| **Death** | enoxaparin vs dalteparin | 1.50(0.41,5.46) | enoxaparin:27.5 |
|  |  |  | **dalteparin:67.3** |

RR, relative risk; 95%CI, 95% confidence interval.VTE, venous thrombus embolism; DVT, deep vein thrombosis.

**Table S10. Subgroup analyses of RCTs’ patients >65 years old**

| **Outcome** | **Comparison** | **RR（95%CI）** | **SUCRA** |
| --- | --- | --- | --- |
| **VTE or VTE related death** | cetoparin vs enoxaparin | 1.17(0.20,6.83) | certoparin:44.7 |
|  | cetoparin vs tinzaparin | 1.62(0.18,14.67) | enoxaparin:52.8 |
|  | certoparin vs nadroparine | 0.62(0.02,16.99) | **tinzaparin:68.1** |
|  | enoxaparin vs tinzaparin | 1.38(0.29,6.60) | nadroparine:34.2 |
|  | enoxaparin vs nadroparine | 0.53(0.03,9.85) |  |
|  | tinzaparin vs nadroparine | 0.38(0.02,9.52) |  |
| **Clinically relevant bleeding** | cetoparin vs enoxaparin | 1.35(0.45,4.02) | cetoparin:22.9 |
|  | cetoparin vs tinzaparin | 1.57(0.38,6.65) | enoxaparin:38.0 |
|  | cetoparin vs nadroparine | 7.72(0.28,213.60) | tinzaparin:50.3 |
|  | certoparin vs dalteparin | 1.72(0.47,6.23) | **nadroparine:85.2** |
|  | enoxaparin vs tinzaparin | 1.17(0.41,3.35) | dalteparin:58.1 |
|  | enoxaparin vs nadroparine | 5.73(0.24,137.39) |  |
|  | enoxaparin vs dalteparin | 1.27(0.54,2.99) |  |
|  | tinzaparin vs nadroparine | 4.91(0.18,134.02) |  |
|  | tinzaparin vs dalteparin | 1.09(0.31,3.83) |  |
|  | nadroparine vs dalteparin | 0.22(0.01,5.74) |  |
| **Death** | certoparin vs enoxaparin | 1.08(0.57,2.05) | certoparin:25.9 |
|  | certoparin vs tinzaparin | 1.83(0.81,4.13) | enoxaparin:30.7 |
|  | certoparin vs nadroparine | 2.02(0.557.39) | **tinzaparin:83.6** |
|  | certoparin vs dalteparin | 1.24(0.64,2.41) | nadroparine:78.5 |
|  | enoxaparin vs tinzaparin | 1.69(0.95,3.01) | dalteparin:53.9 |
|  | enoxaparin vs nadroparine | 1.87,(0.59,5.97) |  |
|  | enoxaparin vs dalteparin | 1.15(0.82,1.60) |  |
|  | tinzaparin vs nadroparine | 1.11(0.31,3.92) |  |
|  | tinzaparin vs dalteparin | 0.68(0.37,1.24) |  |
|  | nadroparine vs dalteparin | 0.61(0.19,1.99) |  |

RR, relative risk; 95%CI, 95% confidence interval.VTE, venous thrombus embolism; DVT, deep vein thrombosis.

**Table S11. Subgroup analyses of RCTs’ patients >70 years old**

| **Outcome** | **Comparison** | **RR（95%CI ）** | **SUCRA** |
| --- | --- | --- | --- |
| **VTE or VTE related death** | cetoparin VS enoxaparin | 1.31(0.23,7.36) | cetoparin：43.9 |
|  | cetoparin vs tinzaparin | 1.62(0.19,14.00) | enoxaparin：59.3 |
|  | cetoparin vs nadroparine | 0.62(0.02,16.28) | nadroparine：32.6 |
|  | enoxaparin vs tinzaparin | 1.24(0.26,5.81) | **tinzaparin: 67.4** |
|  | enoxaparin vs nadroparine | 0.47(0.03,9.23) |  |
|  | tinzaparin vs nadroparine | 0.38(0.02,9.17) |  |
| **Clinically relevant bleeding** | cetoparin vs enoxaparin | 1.34(0.42,4.29) | cetoparin:24.7 |
|  | cetoparin vs tinzaparin | 1.59(0.35,7.20) | enoxaparin:39.1 |
|  | cetoparin vs nadroparine | 7.72(0.27,223.20) | tinzaparin:52.5 |
|  | cetoparin vs dalteparin | 1.60(0.31,8.25) | **nadroparine:84.2** |
|  | enoxaparin vs tinzaparin | 1.19(0.39,3.61) | dalteparin:51.7 |
|  | enoxaparin vs nadroparine | 5.77(0.23,141.98) |  |
|  | enoxaparin vs dalteparin | 1.20(0.33,4.29) |  |
|  | tinzaparin vs nadroparine | 4.87(0.17,138.10) |  |
|  | tinzaparin vs dalteparin | 1.01(0.20,5.00) |  |
|  | nadroparine vs dalteparin | 0.21(0.01,6.24) |  |
| **Death** | cetoparin vs enoxaparin | 1.08(0.57,2.05) | cetoparin:25.7 |
|  | cetoparin vs tinzaparin | 1.83(0.81,4.13) | enoxaparin:31.0 |
|  | cetoparin vs nadroparine | 2.02(0.55,7.39) | **tinzaparin:83.8** |
|  | cetoparin vs dalteparin | 1.24(0.64,2.41) | nadroparine:78.6 |
|  | enoxaparin vs tinzaparin | 1.69(0.95,3.01) | dalteparin:54.0 |
|  | enoxaparin vs nadroparine | 1.87(0.58,5.97) |  |
|  | enoxaparin vs dalteparin | 1.15(0.82,1.60) |  |
|  | tinzaparin vs nadroparine | 1.11(0.31,3.92) |  |
|  | tinzaparin vs dalteparin | 0.68(0.37,1.24) |  |

RR, relative risk; 95%CI, 95% confidence interval.VTE, venous thrombus embolism; DVT, deep vein thrombosis.

**Table S12. Subgroup analyses of RCTs’ patients >75 years old**

| **Outcome** | **Comparison** | **RR（95%CI）** | **SUCRA** |
| --- | --- | --- | --- |
| **Clinically relevant bleeding** | enoxaparin vs dalteparin | 1.27(0.32,5.04) | enoxaparin:33.8 |
|  |  |  | **dalteparin:59.1** |
| **Death** | enoxaparin vs dalteparin | 1.15(0.82,1.60) | enoxaparin:38.4 |
|  |  |  | **dalteparin:81.9** |

RR, relative risk; 95%CI, 95% confidence interval.VTE, venous thrombus embolism; DVT, deep vein thrombosis.

**Table S13.** **Subgroup analyses of RCTs’ sample size larger than 100**

| **Outcome** | **Comparison** | **RR（95%CI ）** | **SUCRA** |
| --- | --- | --- | --- |
| **VTE or VTE related death** | cetoparin vs enoxaparin | 1.08(0.17,6.99) | cetoparin:39.8 |
|  | cetoparin vs tinzaparin | 2.03(0.15,27.31) | enoxaparin:40.4 |
|  | enoxaparin vs tinzaparin | 1.89(0.26,14.04) | **tinzaparin:72.5** |
| **Clinically relevant bleeding** | cetoparin vs enoxaparin | 1.48(0.54,4.04) | cetoparin:23.2 |
|  | cetoparin vs tinzaparin | 1.43(0.36,5.61) | enoxaparin:55.8 |
|  | cetoparin vs dalteparin | 1.72(0.53,0.62) | tinzaparin:51.0 |
|  | enoxaparin vs tinzaparin | 1.00(0.37,2.69) | **dalteparin:69.7** |
|  | enoxaparin vs dalteparin | 1.20(0.59,2.46) |  |
|  | tinzaparin vs dalteparin | 1.21(0.36,4.10) |  |
| **Death** | cetoparin vs enoxaparin | 1.11(0.58,2.11) | cetoparin:27.8 |
|  | cetoparin vs tinzaparin | 1.94(0.85,4.46) | enoxaparin:38.5 |
|  | cetoparin vs dalteparin | 1.24(0.64,2.41) | **tinzaparin:95.3** |
|  | enoxaparin vs tinzaparin | 1.75(0.96,3.20) | dalteparin:61.1 |
|  | enoxaparin vs dalteparin | 1.12(0.80,1.57) |  |
|  | tinzaparin vs dalteparin | 0.64(0.34,1.20) |  |

RR, relative risk; 95%CI, 95% confidence interval.VTE, venous thrombus embolism; DVT, deep vein thrombosis.

**Table S14. Subgroup analyses of RCTs followed-up shorter than 60days**

| **Outcome** | **Comparison** | **RR（95%CI ）** | **SUCRA** |
| --- | --- | --- | --- |
| **VTE or VTE related death** | cetoparin vs enoxaparin | 0.87(0.12,6.10) | **cetoparin：49.1** |
|  |  |  | enoxaparin:34.4 |
| **Clinically relevant bleeding** | cetoparin vs enoxaparin | 1.25(0.37,4.25) | cetoparin:30.4 |
|  |  |  | **enoxaparin:46.7** |
| **Death** | cetoparin vs enoxaparin | 1.03(0.48,2.21) | cetoparin:44.4 |
|  |  |  | **enoxaparin:48.3** |

RR, relative risk; 95%CI, 95% confidence interval.VTE, venous thrombus embolism; DVT, deep vein thrombosis.

**Table S15. Subgroup analyses of RCTs followed-up longer than 60 days.**

| **Outcome** | **Comparison** | **RR（95%CI ）** | **SUCRA** |
| --- | --- | --- | --- |
| **VTE or VTE related death** | enoxaparin vs tinzaparin | 0.99(0.25,3.94) | **enoxaparin:71.2** |
|  | enoxaparin vs nadroparine | 0.37(0.02,5.60) | tinzaparin:65.6 |
|  | tinzaparin vs nadroparine | 0.37(0.02,6.63) | nadroparine:27.0 |
| **Clinically relevant bleeding** | enoxaparin vs tinzaparin | 0.80(0.48,1.33) | enoxaparin:63.9 |
|  | enoxaparin vs nadroparine | 4.21(0.21,85.77) | tinzaparin:28.5 |
|  | enoxaparin vs dalteparin | 0.96(0.65,1.43) | **nadroparine:83.4** |
|  | tinzaparin vs nadroparine | 5.28(0.25,110.23) | dalteparin:56.2 |
|  | tinzaparin vs dalteparin | 1.21(0.69,2.09) |  |
|  | nadroparine vs dalteparin | 0.23(0.01,4.68) |  |
| **Death** | enoxaparin vs tinzaparin | 1.55(0.71,3.37) | enoxaparin:63.9 |
|  | enoxaparin vs nadroparine | 1.72(0.48,6.13) | tinzaparin:28.5 |
|  | enoxaparin vs dalteparin | 1.05(0.57,1.95) | **nadroparine:83.4** |
|  | tinzaparin vs nadroparine | 1.11(0.31,3.92) | dalteparin:56.2 |
|  | tinzaparin vs dalteparin | 0.68(0.37,1.24) |  |
|  | nadroparine vs dalteparin | 0.61(0.19,1.99) |  |

RR, relative risk; 95%CI, 95% confidence interval.VTE, venous thrombus embolism; DVT, deep vein thrombosis.

**Table S16. Subgroup analyses of multi-center RCTs.**

| **Outcome** | **Comparison** | **RR（95%CI）** | **SUCRA** |
| --- | --- | --- | --- |
| **VTE or VTE related death** | certoparin vs enoxaparin | 1.20(0.19,7.70) | certoparin:38.1 |
|  | certoparin vs tinzaparin | 2.03(0.16,26.66) | enoxaparin:47.3 |
|  | enoxaparin vs tinzaparin | 1.70(0.23,12.53) | **tinzaparin:71.4** |
| **Clinically relevant bleeding** | certoparin vs enoxaparin | 1.36(0.46,4.03) | certoparin:26.4 |
|  | certoparin vs tinzaparin | 1.43(0.33,6.24) | enoxaparin:46.6 |
|  | certoparin vs dalteparin | 1.72(0.48,6.17) | tinzaparin:52.3 |
|  | enoxaparin vs tinzaparin | 1.05(0.34,3.22) | **dalteparin:70.5** |
|  | enoxaparin vs dalteparin | 1.26(0.54,2.93) |  |
|  | tinzaparin vs dalteparin | 1.21(0.32,4.48) |  |
| **Death** | certoparin vs enoxaparin | 1.04(0.50,2.17) | certoparin:30.3 |
|  | certoparin vs tinzaparin | 1.94(0.83,4.54) | enoxaparin:31.9 |
|  | certoparin vs dalteparin | 1.24(0.62,2.46) | **tinzaparin:94.5** |
|  | enoxaparin vs tinzaparin | 1.86(0.92,3.74) | dalteparin:60.5 |
|  | enoxaparin vs dalteparin | 1.19(0.73,1.94) |  |
|  | tinzaparin vs dalteparin | 0.64(0.33,1.22) |  |

RR, relative risk; 95%CI, 95% confidence interval.VTE, venous thrombus embolism; DVT, deep vein thrombosis.

**Table S17. Subgroup analyses of single center RCTs.**

| **Outcome** | **Comparison** | **RR（95%CI）** | **SUCRA** |
| --- | --- | --- | --- |
| **VTE or VTE related death** | enoxaparin vs tinzaparin | 1.37(0.08,22.20) | enoxaparin:41.9 |
|  | enoxaparin vs nadroparine | 0.79(0.03,19.98) | **tinzaparin:56.1** |
|  | tinzaparin vs nadroparine | 0.58(0.01,24.91) | nadroparine:39.0 |
| **Clinically relevant bleeding** | enoxaparin vs tinzaparin | 4.00(0.14,110.63) | enoxaparin:16.7 |
|  | enoxaparin vs nadroparine | 11.61(0.25,548.26) | tinzaparin:60.8 |
|  | tinzaparin vs nadroparine | 2.90(0.03,243.79) | **arine:79.1** |
| **Death** | enoxaparin vs tinzaparin | 0.75(0.11,5.21) | enoxaparin:53.1 |
|  | enoxaparin vs nadroparine | 1.54(0.41,5.81) | tinzaparin:36.7 |
|  | tinzaparin vs nadroparine | 2.04(0.24,17.32) | **nadroparine:78.6** |

RR, relative risk; 95%CI, 95% confidence interval.VTE, venous thrombus embolism; DVT, deep vein thrombosis.

**Table S18. Subgroup analyses of RCTs before 2010.**

| **Outcome** | **Comparison** | **RR（95%CI）** | **SUCRA** |
| --- | --- | --- | --- |
| **VTE or VTE related death** | enoxaparin vs tinzaparin | 1.20(0.10,15.07) | enoxaparin:43.0 |
|  | enoxaparin vs nadroparine | 0.70(0.04,13.32) | **tinzaparin:54.6** |
|  | tinzaparin vs nadroparine | 0.58(0.01,24.55) | nadroparine:37.9 |
| **Clinically relevant bleeding** | enoxaparin vs tinzaparin | 1.34(0.13,13.49) | enoxaparin:57.2 |
|  | enoxaparin vs nadroparine | 3.90(0.19,80.42) | tinzaparin:55.6 |
|  | enoxaparin vs dalteparin | 0.88(0.56,1.39) | **nadroparine:79.6** |
|  | tinzaparin vs nadroparine | 2.90(0.07,127.98) | dalteparin:42.5 |
|  | tinzaparin vs dalteparin | 0.66(0.06,6.71) |  |
|  | nadroparine vs dalteparin | 0.23(0.01,4.72) |  |
| **Death** | enoxaparin vs tinzaparin | 0.87(0.14,5.39) | enoxaparin:44.9 |
|  | enoxaparin vs nadroparine | 1.78(0.55,5.69) | tinzaparin:39.2 |
|  | enoxaparin vs dalteparin | 1.09(0.77,1.53) | **nadroparine:80.0** |
|  | tinzaparin vs nadroparine | 2.04(0.24,17.32) | dalteparin:58.6 |
|  | tinzaparin vs dalteparin | 1.25(0.20,7.78) |  |
|  | nadroparine vs dalteparin | 0.61(0.19,1.99) |  |

RR, relative risk; 95%CI, 95% confidence interval.VTE, venous thrombus embolism; DVT, deep vein thrombosis.

**Table S19. Subgroup analyses of RCTs after 2010.**

| **Outcome** | **Comparison** | **RR（95%CI）** | **SUCRA** |
| --- | --- | --- | --- |
| **VTE or VTE related death** | certoparin vs enoxaparin | 1.50(0.19,11.71) | certoparin:36.8 |
|  | certoparin vs tinzaparin | 2.03(0.14,29.92) | enoxaparin:58.1 |
|  | enoxaparin vs tinzaparin | 1.36(0.15,12.02) | **tinzaparin:67.6** |
| **Clinically relevant bleeding** | certoparin vs enoxaparin | 1.03(0.31,3.43) | certoparin:35.4 |
|  | certoparin vs tinzaparin | 1.43(0.29,6.89) | enoxaparin:30.1 |
|  | enoxaparin vs tinzaparin | 1.38(0.40,4.78) | **tinzaparin:62.8** |
| **Death** | certoparin vs enoxaparin | 0.81(0.29,2.20) | certoparin:41.8 |
|  | certoparin vs tinzaparin | 1.94(0.62,6.07) | enoxaparin:17.6 |
|  | enoxaparin vs tinzaparin | 2.41(0.90,6.42) | **tinzaparin:92.1** |

RR, relative risk; 95%CI, 95% confidence interval.VTE, venous thrombus embolism; DVT, deep vein thrombosis.

**Table S20. Subgroup analyses of RCTs of enoxaparin were removed.**

| **Outcome** | **Comparison** | **RR（95%CI）** | **SUCRA** |
| --- | --- | --- | --- |
| **VTE or VTE related death** | cetoparin vs tinzaparin | 1.83(0.81,4.13) | **cetoparin:71.2** |
|  | cetoparin vs nadroparine | 0.62(0.06,6.75) | tinzaparin:65.6 |
|  | tinzaparin vs nadroparine | 0.34(0.03,4.01) | nadroparine:27.0 |
| **Clinically relevant bleeding** | cetoparin vs tinzaparin | 1.46(0.83,2.58) | cetoparin:5.5 |
|  | cetoparin vs nadroparine | 7.72(0.38,158.82) | tinzaparin:46.0 |
|  | cetoparin vs dalteparin | **1.76(1.10,2.82)** | **nadroparine:86.4** |
|  | tinzaparin vs nadroparine | 5.28(0.25,110.20) | dalteparin:70.1 |
|  | tinzaparin vs dalteparin | 1.21(0.69,2.09) |  |
|  | nadroparine vs dalteparin | 0.23(0.01,4.68) |  |
| **Death** | cetoparin vs tinzaparin | 1.83(0.81,4.13) | cetoparin:22.5 |
|  | cetoparin vs nadroparine | 2.02(0.55,7.39) | **tinzaparin:80.9** |
|  | cetoparin vs dalteparin | 1.24(0.64,2.41) | nadroparine:76.8 |
|  | tinzaparin vs nadroparine | 1.11(0.31,3.92) | dalteparin:47.7 |
|  | tinzaparin vs dalteparin | 0.68(0.37,1.24) |  |
|  | nadroparine vs dalteparin | 0.61(0.19,1.99) |  |

RR, relative risk; 95%CI, 95% confidence interval.VTE, venous thrombus embolism; DVT, deep vein thrombosis.
